# 

**Published:** 1848-04

**Authors:** 


### GRADUATES

OF

### JEFFERSON MEDICAL COLLEGE OF PHILADELPHIA,

### MARCH, 1848.

---

*At a Public Commencement held on the 29th of March*, 1848, *the Degree of* DOCTOR OF MEDICINE *was conferred on the following gentlemen by the* REV. EZRA STILES ELY, D. D., *President protempore in the absence of the* REV. ASHBEL GREEN, D. D., L. L. D., *President of the Institution, after which a charge to the Graduates was delivered by* PROFESSOR PANCOAST.

| Name. | State. | Subject of Thesis. |
|---|---|---|
| Alexander, Gerard | Kentucky, | Syphilis. |
| Anderson, John B. | Virginia. | Hypertrophy of the Heart. |
| Arnold, Edmund S. F. | England. | Pneumothorax. |
| Ayer, Francis B. | New Hampshire. | Typhoid Fever. |
| | | |
| Baer, Caleb D. | Maryland. | Hydrocele. |
| Bainbridge, Eusebius C. | Kentucky. | Prolapsus Uteri. |
| Banks, William | Alabama. | Cold Stage of Intermittent Fever. |
| Baxley, Jabez B. | South Carolina. | Typhus Fever. |
| Bennett, Trumbull L. | New York. | Phthisis Pulmonalis. |
| Benson, Wirt F. | Tennessee. | Physiology of Respiration. |
| Bibb, William E. | Virginia. | Opium. |
| Birch, David | Ireland. | Nutriment of Fœtus. |
| Birdsell, Sylvester | Pennsylvania. | Cardiac Dropsy. |
| Blain, Hamilton L. | Virginia. | Dysentery. |
| Bolling, Lenæus | Virginia. | Acute Gastritis. |
| Bolton, James N. | Georgia. | Typhoid Fever. |
| Bourn, Richard W. | Missouri. | Datura Stramonium. |
| Bournonville, A., M.D. | Pennsylvania. | (Ad eundem.) |
| Bowen, James W. | Maryland. | Bilious Remittent Fever. |
| Brodnax, Robert | North Carolina. | Circulation of the Blood. |
| Brookbank, John W. | Iowa. | Intermittent Fever. |
| Brown, Henry James, M.D. | Pennsylvania | Tetanus. |
| Brass, Andrew J. | Pennsylvania. | Eclampsia Infantum. |
| Brown, John T. | Pennsylvania. | Acute Dysentery. |
| Bruner, William H. | Pennsylvania. | Glands of Brunner. |
| Buck, Jonathan | Mississippi. | Fever. |
| | | |
| Cable, Josiah C. | Ohio. | Physical Diagnosis. |
| Cahall, Thomas | Delaware. | Epidemic Erysipelas as it occurred at Dover, Del., in 1847. |
| Cameron, J. Walton | Virginia. | Pertussis. |
| Carr, William W. | Georgia. | Pneumonia. |
| Chambers, John M. Duncan | Pennsylvania. | Typhoid Fever. |
| Chancellor, James E. | Virginia. | Digestion. |
| Churchman, Vincent T. | Virginia. | Differential Diagnosis of Typhus and Typhoid Fever. |
| Clark, George W. | Virginia. | Peritonitis. |
| Clements, L. Morgan | Alabama. | Acute Pleuritis. |

| | | |
|---|---|---|
| Collet, Mark Wilkes | Pennsylvania. | Changes of the Blood in disease. |
| Connalley, Reps | Virginia. | Acute Hydrocephalus. |
| Crouse, George Jackson | Pennsylvania. | Febris Remittens. |
| | | |
| Davidson, Charles Edward | Virginia. | Puerperal Fever. |
| Davis, John, Jr. | Virginia. | Hematology. |
| Dennis, William H. | Virginia. | Cholera Infantum. |
| Deupree, William J. | Mississippi. | Congestive Fever. |
| Dice, Reuben B. | Virginia. | Acute Endo-gastritis. |
| Dixon, Lucius | Virginia. | Diseases of the Urinary bladder. |
| Dodd, Robert J., Jr. | Pennsylvania. | Process of Digestion. |
| Doxey, John L. | Mississippi. | Gonorrhœa. |
| Drayton, Edward F. | Pennsylvania. | Curvatures of the Spine. |
| | | |
| Fitzgerald, Edmund | Georgia. | Pathology of Inflammation. |
| Foard, Andrew Jackson | Georgia. | Rheumatism. |
| Folwell, Joseph N. | Kentucky. | Acute Dysentery. |
| Fort, Moses T. | Georgia, | Moral prophylaxis and treatment of diseases. |
| Frick, William S. | Pennsylvania. | Pharmaceutical Inconsistencies. |
| | | |
| Gamble, George M. | Pennsylvania. | Conception. |
| Garnett, James H. | Virginia. | Differential Diagnosis of Typhus and Typhoid Fever. |
| Gauthreaux, Joseph Justin | Louisiana. | Typhus Fever. |
| Gilbert, Julius Cæsar | Georgia. | Yellow Fever. |
| Glass, Samuel | Ohio. | Pneumonia. |
| Green, James S. | North Carolina. | Constipation and its effects. |
| Gresham, Sterling A. | Georgia. | Cholera Infantum. |
| Guild, La Fayette | Alabama. | Healing of wounds. |
| Gwyn, James D. | North Carolina. | Vis Medicatrix Naturæ. |
| | | |
| Habersham, Stephen Elliott | South Carolina. | Inguinal Hernia. |
| Halley, Samuel H. | Virginia. | Scarlatina. |
| Hamilton, James | Pennsylvania. | Pathology of the Human Solids. |
| Harden, Robert Raymond | Georgia. | Acute Gastritis. |
| Harper, John P. | Missouri. | Acute Peritonitis. |
| Harris, Marcus A. | Virginia. | Diagnosis. |
| Hayes, Pliny H. | New York. | Physiological Anatomy of the skin |
| Henderson, William J. | Pennsylvania. | Hepatitis. |
| Herr, Henry F. | Pennsylvania. | Fecundation. |
| Hill, Alonzo A. F. | Georgia. | Hereditary Transmission. |
| Hillsman, William H. | Virginia. | Pneumonia. |
| Hobson. Samuel A. | Virginia. | Inguinal and Femoral Hernia. |
| Hore, Walter | Virginia. | Dysentery. |
| Hottenstein, Cyrus D. | Pennsylvania. | Amenorrhœa. |
| Houtz, Abraham | Ohio. | Typhoid Fever. |
| Howard, Nelson W. | New York. | Quackery. |
| Howell, George H. | Pennsylvania. | Pneumonia. |
| Humphreys, Benjamin W. | Tennessee. | Natural Indications. |
| Hunter, William R. | Maine. | Sulphuric Ether. |
| | | |
| Isbell, Abner C. | Virginia. | Acute Articular Rheumatism. |
| Jackson, Samuel | Pennsylvania. | Opium. |
| Jenkins, Paul F. | South Carolina. | Gastritis. |
| Jenkins, Robert C. | Virginia. | Scarlatina. |
| Jessup, John J. | New Jersey. | The Tampon. |
| Jeter, John W. | Virginia. | Hysteria. |
| Jones, Daniel W. | Maryland. | Cutaneous System. |

| | | |
|---|---|---|
| Jones, Robert Lewis | Mississippi. | Animal Calorification. |
| Jordan, Watson P. | Virginia. | Bilious Remittent Fever. |
| Kendrick, Oscar C. | Ohio. | Action of Ergot on the vitality of the Blood. |
| Knapp, Franklin A. | New York. | Menstruation. |
| Koontz, George H. H. | Virginia. | Tic Douleureux. |
| Ladd, Horace | Pennsylvania. | Acute Pneumonia. |
| Leake, Virginius | Tennessee. | Vis Medicatrix Naturæ. |
| Leatherbury, Edward R. | Virginia. | Pneumonia. |
| Levis, Richard J. | Pennsylvania. | Influence of the mind on Disease. |
| Lindsey, Hugh N. | Mississippi. | Chylosis. |
| Linn, William H. | New Jersey. | Intermittent Fever. |
| Long, John Wesley | North Carolina. | Menstruation. |
| Long, Reuben K. | Virginia. | Asphyxia. |
| Lothrop, James E. | New Hampshire. | Pneumonia. |
| Luther, Martin | Pennsylvania. | Acute Peritonitis. |
| McClure, Henry | Pennsylvania. | Intermittent Fever. |
| McIlvaine, Robert H. | North Carolina. | Qualifications of a Physician. |
| McNail, Thomas A. | Tennessee. | Acute Hepatitis. |
| Mackenzie, Thomas G. | Virginia. | Use of Sulphuric Ether. |
| Marsden, John H. | Pennsylvania. | Management of Lying-in Women. |
| Marshall, Theophilus O. | Virginia. | Cathartics and their Therapeutical Effects. |
| Mason, Edmunds | Alabama. | Typhoid, contrasted with Typhus Fever. |
| Merinar, William H. | Mississippi. | Yellow Fever. |
| Moore, William I. | New Jersey. | Iodine. |
| Moss, George W. | Missouri. | Remittent Fever of Northern Missouri. |
| Moultrie, Alonzo C. | Georgia. | Delirium Tremens. |
| Murphy, Cornelius T. | North Carolina. | Rubeola. |
| Murphy, John A. | Pennsylvania. | Peritonitis. |
| Nash, John W. | Virginia. | Asthma. |
| Neblett, Sterling, Jr. | Virginia. | Acute and Chronic Peritonitis. |
| Neff, Peter D. | Pennsylvania. | Asphyxia. |
| Nicholas, John Thomas | South Wales. | Lithuria. |
| O'Donnell, William | Pennsylvania. | Abortion. |
| Oldham, Robert H. | Tennessee. | Typhus Fever. |
| Osborne, Edward A. | New Jersey. | Typhoid Fever. |
| Owen, Edward | Pennsylvania. | Typhoid Fever. |
| Palmer, Charles | New Hampshire. | Apoplexy. |
| Parker, Edward H. | Massachusetts. | Diabetes Mellitus. |
| Patterson, Theophilus | New Jersey. | Dyspepsia. |
| Peters, Samuel Adams | Missouri. | Amenorrhœa. |
| Pettus, Luther C. | Virginia. | Chlorosis. |
| Phillips, Thomas A. | Mississippi. | Medical Empiricism. |
| Philbrick, Samuel R. | Massachusetts. | Principles of Diognosis. |
| Preston, Alexander R., M:D. | Virginia. | (Ad eundem.) |
| Prince, William E. | Virginia. | Typhoid Fever. |
| Puryear, Richard R. | Virginia. | Carbonic Acid. |
| Ramsay, G. Randolph | Georgia. | Dyspepsia. |
| Ramsay, James Graham | North Carolina. | Congestive Fever. |
| Rand, B. Howard | Pennsylvania. | Animal Charcoal as an Antidote. |
| Reilly, Paul Jones | Missouri. | Duties of the Accoucheur. |

| | | |
|---|---|---|
| Richardson, William L. | Pennsylvania. | Scarlet Fever. |
| Robb, John P. | Virginia. | Dysentery. |
| Robertson, Edwin J. | Virginia. | Therapeutical Agency of Sulphate of Quinia. |
| Robson, George T. | Virginia. | Remittent Fever. |
| Rogers, William Egbert | Tennessee. | Ammonia. |
| Rooke, Levi | Pennsylvania. | Phrenology. |
| | | |
| Sample, John | Pennsylvania. | Acute Gastritis. |
| Scott, Samuel | Pennsylvania. | Pathology of Scarlatina. |
| Seltzer, John Horace | Pennsylvania. | Cynanche Trachealis. |
| Service, Lecky M. | Pennsylvania. | Adaptation of the Hand to the Arts and Sciences. |
| Smith, Elliott Iverson | Georgia. | Emansio Mensium. |
| Sutton, James L. | Pennsylvania, | Dropsy, |
| Sutton, Lewis | Pennsylvania. | Cynanche Trachealis. |
| | | |
| Taylor, George T. | New Jersey. | Scarlatina. |
| Taylor, James M. | Pennsylvania. | Continued Fever. |
| Taylor, Leonidas C. | North Carolina. | Diagnosis of Pregnancy. |
| Terhune, Archibald A. | Georgia. | Moving Powers of the Blood. |
| Tharp, Jonathan | Delaware. | Pneumonia. |
| Tharp, William H. | Tennessee. | Intermittent Fever. |
| Thomas, John R. | Pennsylvania. | Pneumonia. |
| Thornton, Wm. P., M. D. | Mississippi. | (Ad eundem.) |
| Timberlake, Phillip | Virginia. | Reproduction. |
| Tobias, John F. | Pennsylvania. | Acute Peritonitis. |
| Todd, Alexander H. | Maryland. | Typhus Fever. |
| Toombs, Robert E. | Georgia. | Gastritis. |
| Turner, James | Pennsylvania. | Acute Hepatitis. |
| | | |
| Urquhart, Thomas H. | Virginia. | Acute Dysentery. |
| | | |
| Vaughan, Thomas B. | Alabama. | Colica Pictonum. |
| | | |
| Wallace, Robert B. | Alabama. | Endo-gastritis. |
| Walsh, William F. | Tennessee. | Erysipelas. |
| Walton, Lewis I. | Virginia. | Carbonic Acid Gas. |
| Welch, William A. | Alabama. | Peripneumony. |
| Whitaker, Benjamin F. | North Carolina. | Blood-letting. |
| White, G. Jefferson | Virginia. | Scarlatina. |
| Williams, Jr., William | Virginia. | Epidemic Fever that prevailed in Virginia, during the Autumn of 1845. |
| Wilson, Andrew J. | Virginia. | Tight Lacing. |
| Wilson, James A. | Pennsylvania. | Scarlatina. |
| Wimbish, James A. | Virginia. | Hydrogen. |
| Womble, Pembroke M. | Virginia. | Measles. |
| Wright, John | Pennsylvania | Puerperal Fever. |
| | | |
| Zimmerman, Reuben P. | Missouri. | Medicine. |

Total, 178.

R. M. HUSTON, M. D., Dean.

